# Response to the Letter to the Editor: “COVID-19 Infection Risk, Elective Arthroplasty and Surgical Complications, and COVID-19 Vaccination: Correspondence”

**DOI:** 10.1016/j.artd.2022.10.013

**Published:** 2022-11-04

**Authors:** Peyman Mirghaderi, Reza Mirghaderi, S.M. Javad Mortazavi

**Affiliations:** Joint Reconstruction Research Center, Tehran University of Medical Sciences, Tehran, Iran; Students' Scientific Research Center (SSRC), Tehran University of Medical Sciences, Tehran, Iran; Joint Reconstruction Research Center, Tehran University of Medical Sciences, Tehran, Iran

We would like to thank both Dr. Mungmunpuntipantip for his precise comments on our article [1] and the Editor-in-Chief of *Arthroplasty Today* for giving us the opportunity to respond. The comments were read with specific interest, and we attempted to respond to our colleague's questions, acknowledging the limitations of the study which could also result in misinterpretation of the results. In the following paragraph, we describe the 3 consecutive studies we published in 2022 about COVID-19 infection after total joint arthroplasty (TJA).

We conducted a prospective study in which we followed up TJA patients following discharge for symptoms of COVID-19 and then confirmed the diagnosis using reverse transcription polymerase chain reaction. In 3 published studies, the rate and risk factors of symptomatic COVID-19 were described. First, we evaluated unvaccinated patients who underwent an elective TJA between April 2020 and April 2021 and found that their COVID-19 infection rate was 2.4% (18/755), which was not greater than that of the general population (2.2%, *P* > .05) [2]. As a result of our study, we concluded that resuming elective TJA surgeries in the prevaccine era was not free of risks. Both surgeons and patients should be aware of these risks, and perioperative safety protocols should be adhered to strictly. In the second study, we compared the incidence of COVID-19 infection following urgent vs elective total hip arthroplasty among unvaccinated individuals between April 2020 and August 2021 [3]. The COVID-19 rate among elective cases (1.4%, 5/340) and traumatic cases (3.3%, 3/91) did not differ statistically (*P* = .24). The hypothesis that urgent traumatic cases are at greater risk due to less severe prevention measures failed, and research found a similar risk of infection during the perisurgery period in both elective and urgent total hip arthroplasty cases. In the third and present study, we repeat the first study among vaccinated patients between October 2021 and March 2022 [1]. In vaccinated individuals, the rate of symptomatic COVID-19 within 1 month of elective arthroplasty was 3.9% (38/962), which was not statistically different from the unvaccinated cohort rate (*P* = .07). The 90-day surgical complications of TJA were similar between the vaccinated and unvaccinated groups (*P* > .05) [1]. In conclusion, vaccination does not guarantee that a patient will not contract COVID-19 following an arthroplasty surgery, and reasonable precautions should be taken perioperatively to prevent the infection.

We agree with the author's statement regarding the importance of asymptomatic COVID-19 in these cases. COVID-19 asymptomatic cases carry the virus, can infect other patients on the ward, and can even become symptomatic during the critical recovery period following the surgery [4]. COVID-19-positive cases were found to have inferior surgical outcomes and a higher mortality rate [5]. Therefore, performing strict testing might be a reasonable approach, and in 1 of our study cities, Isfahan, we performed preoperative testing. However, due to limitations in our low-income health-care system, complete laboratory testing was not available in all centers, especially in not-for-profit public hospitals. Additionally, we should be aware of the high rate of false-negative results associated with the reverse transcription polymerase chain reaction test (up to 54%) and should not rely exclusively on it for diagnostic purposes [6].

Additionally, we agree with the author's comment that many factors affect patients' immunization, including comorbidity, new virus variants, and the type and manner of vaccine administration. Still, the vaccine was a fantastic idea, which had a significant impact on reducing COVID-19's dire consequences [7]. They recommended that laboratory tests be conducted on vaccinated patients or patients with previous exposures to COVID-19 to ensure that they are immunized. A number of immunological deficiencies may hinder vaccine effectiveness, and preoperative laboratory evaluations are necessary for some high-risk patients in order to prevent further complications. Immunocompromised patients, such as those with hematological malignancies and organ transplants, are at greater risk of developing severe COVID-19 and developing less antibody protection [8,9]. It is possible that the vaccines are adequate, but not in cases of immunodeficiency. Nevertheless, we believe that performing immunological testing before a TJA surgery on vaccinated patients is not cost-effective in our low-income country with significant health-care deficiencies, except for patients who are at high risk or suspicious [10]. Also, Food and Drug Administration recommends against assessing immunity and antibody levels following COVID-19 vaccination [11]. A misinterpretation of antibody results can result in people taking fewer precautions against severe acute respiratory syndrome coronavirus 2 (SARS-CoV-2) exposure. There is no clear correlation among the results of a SARS-CoV-2 antibody test, the need for a COVID-19 vaccine or booster, or the effectiveness of a vaccine. Moreover, some SARS-CoV-2 antibody tests may not detect the type of antibody produced as a result of vaccination.

Our hope is that this discussion has provided an appropriate response to the comments made by our colleague.

## Conflicts of interest

The authors declare there are no conflicts of interest.

For full disclosure statements refer to https://doi.org/10.1016/j.artd.2022.10.013.

## Appendix A Supplementary Data

Conflict of Interest Statement for Mortazavi

Conflict of Interest Statement for S. P. Mirghaderi

Conflict of Interest Statement for S. R. Mirghaderi
